# Targeted Proteomics Analysis of Staphylococcal Superantigenic Toxins in Menstrual Fluid from Women with Menstrual Toxic Shock Syndrome (mTSS)

**DOI:** 10.3390/toxins14120886

**Published:** 2022-12-19

**Authors:** Marie Courçon, Cédric Badiou, Mathilde Louwagie, Sibyle Etievant, Michel Jaquinod, Gérard Lina, Virginie Brun

**Affiliations:** 1 Univ. Grenoble Alpes, INSERM, CEA, UMR BioSanté U1292, CNRS, CEA, FR2048, 38000 Grenoble, France; 2 CIRI-Centre International de Recherche en Infectiologie, Team Staphylococcal Pathogenesis, Université Claude Bernard Lyon 1, INSERM, U1111, CNRS, UMR5308, ENS Lyon, 69364 Lyon CEDEX 07, France; 3 Centre National de Référence de Staphylocoques-Institut des Agents Infectieux, LBMMS, Hôpital de la Croix-Rousse, Hospices Civils de Lyon, 69317 Lyon CEDEX 04, France; 4 Univ. Grenoble Alpes, CEA, LETI, Clinatec, 38000 Grenoble, France

**Keywords:** proteomics, mass spectrometry, staphylococcal toxic shock syndrome, menstrual fluid, TSST-1

## Abstract

Menstrual toxic shock syndrome (mTSS) is a rare life-threatening febrile illness that occurs in women using intravaginal menstrual protection. It is caused by toxic shock syndrome toxin 1 (TSST-1) produced by *Staphylococcus aureus*, triggering a sudden onset of rash and hypotension, subsequently leading to multiple organ failure. Detecting TSST-1 and *S. aureus* virulence factors in menstrual fluid could accelerate the diagnosis and improve therapeutic management of mTSS. However, menstrual fluid is a highly complex matrix, making detection of bacterial toxins challenging. Here, we present a mass-spectrometry-based proteomics workflow for the targeted, quantitative analysis of four *S. aureus* superantigenic toxins in menstrual fluids (TSST-1, SEA, SEC, and SED). This method was applied to characterize toxin levels in menstrual fluids collected from patients with mTSS and healthy women. Toxins were detectable in samples from patients with mTSS and one healthy donor at concentrations ranging from 0 to 0.46 µg/mL for TSST-1, and 0 to 1.07 µg/mL for SEC. SEA and SED were never detected in clinical specimens, even though many *S. aureus* strains were positive for the corresponding genes. The method presented here could be used to explore toxin production in vivo in users of intravaginal devices to improve the diagnosis, understanding, and prevention of mTSS.

## 1. Introduction

Menstrual toxic shock syndrome (mTSS) is a rare life-threatening disease that occurs in menstruating women using intravaginal protections such as tampons and cups. It is caused by vaginal colonization with *Staphylococcus aureus* producing toxic shock syndrome toxin 1 (TSST-1). mTSS is a sudden febrile illness characterized by rash and hypotension leading to multiple organ failure. Therapeutic management of the disease requires rapid diagnosis and appropriate medical care, including withdrawal of the intravaginal protection, resuscitation, and specific antibiotherapy. This adapted management is often delayed due to the lack of both pathognomonic clinical symptoms in the acute phase and diagnostic tools [1].

Hopefully, not all women hosting TSST-1-producing *S. aureus* strains in their vagina will go on to develop mTSS. Specific conditions are known to promote the risk of developing the disease and its progression: tampon misuse such as a use-time > 6 h or overnight, which promotes intravaginal growth of *S. aureus* and toxin production [2], and a lack of humoral immunity against TSST-1 [3]. However, we still lack information that would allow us to explain why some women develop the disease while others do not.

mTSS pathophysiology is related to the capacity of *S. aureus* to secrete TSST-1 into the vaginal fluid; the toxin can then gain access to the bloodstream, where it triggers the immune system. TSST-1 and other staphylococcal enterotoxins (SEs) are small (19 to 30 kDa) secreted proteins that belong to the superantigen family [4]. They interact in specific ways with antigen-processing cells and T-cells, resulting in an overactivation of T-lymphocytes. This results in a “cytokine storm” responsible for the clinical symptomatology [5,6,7]. Both TSST-1 and SEs are involved in the occurrence of non-menstrual toxic shock syndrome (non-mTSS) that occurs mainly during infections of the skin and soft tissues with *S. aureus*. In contrast, the literature indicates that the vast majority of mTSS cases are caused by TSST-1 [8], even though *S. aureus* strains found in the vagina frequently also possess the genes for SEs [9].

The specific detection of low-abundance proteins such as bacterial toxins and virulence factors in menstrual fluid is extremely challenging. Menstrual fluid is a highly complex and variable sample composed of cervical mucus, vaginal secretions, endometrial tissue, and blood. In 2012, Yang and coworkers [10] identified more than 1000 proteins in menstrual blood, but its true protein content is likely to be much more diverse. Indeed, more recent studies, taking advantage of enhanced instrumentation, have shown that blood is one of the most complex biological matrices, with more than 3000 proteins in plasma [11] and 2650 proteins described in red blood cells [12]. Added to these vast arrays, cervical mucus contains more than 600 proteins [13], and according to transcriptomic analysis (Human protein Atlas) [14], endometrial tissue could express up to 69% (n = 13,898) of all human proteins. In such a complex matrix, detection of bacterial toxins and virulence factors generally requires immunological assays to achieve sufficiently precise targeting and sensitivity. However, these methods present some limitations: (i) they can be hampered by cross-reactivity, especially when targeting highly homologous proteins like SEs; (ii) they suffer from matrix interference, such as the formation of immune complexes involving endogenous anti-toxin antibodies and staphylococcal protein A [15,16]. Consequently, in this specific context, the mass spectrometry (MS)-based detection of proteins presents specific advantages. Firstly, it is a direct and multiplexed analytical method allowing the simultaneous detection and specific identification of proteins based on characteristic peptide sequences. This is a key asset for discrimination between SEs, which can be co-secreted and in some cases share extensive sequence similarity. Secondly, protein complexes are disrupted during sample preparation—which includes protein denaturation and digestion steps before MS analysis, thus matrix interference is avoided. Finally, when combined with isotopically-labeled standards, MS-based proteomics analysis can be used to determine protein concentrations, even in complex biological samples [17].

In this article, we present a mass-spectrometry-based proteomics workflow for the targeted, quantitative analysis of TSST-1, SEA, SEC, and SED superantigenic toxins in menstrual fluids. We applied this method to analyze menstrual fluids from a bank of samples collected from patients with mTSS and healthy women to characterize toxin levels and help decipher the pathogenic role played by these superantigenic toxins in mTSS.

## 2. Results

### 2.1. Development of the Targeted Proteomic Assay

**Figure 1:**
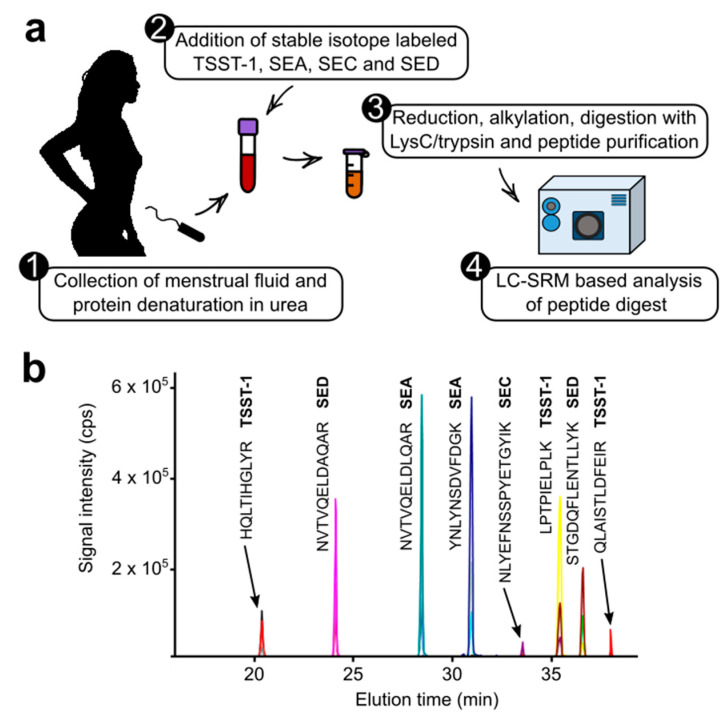
Targeted proteomics analysis of superantigenic toxins present in menstrual fluid. (**a**) Diagram illustrating the experimental workflow involving sample collection and preparation followed by scheduled LC-SRM analysis; (**b**) Extracted ion chromatogram from scheduled LC-SRM, obtained for menstrual fluid spiked with full-length isotope-labeled toxins following protein digestion and analysis. Eight signature peptides corresponding to TSST-1, SEA, SEC, and SED were monitored in their labeled and unlabeled versions.

We started by developing a liquid chromatography-selected reaction monitoring (LC-SRM) method to assay TSST-1, SEA, SEC, and SED (SEB was not included due to safety and regulatory constraints). To do so, signature tryptic peptides for these four toxins were identified, their sequence uniqueness was verified by performing BLAST searches against the UniProt database, and SRM transition lists were generated using Skyline software [18]. Finally, to ensure that these toxins would be specifically detected in menstrual fluids with a high sensitivity, we generated a substitution matrix to assess several pre-analytical preparations and to help develop the LC-SRM analytical workflow. This substitution matrix corresponded to a pool of menstrual fluids from women whose vaginal flora lacked *S. aureus* (i.e., devoid of endogenous toxins). Full-length isotope-labeled versions of the four toxins were synthesized using an in vitro expression system. These standard proteins were spiked into the pool of menstrual fluid to serve as toxin surrogates to allow pre-analytical and analytical optimization. Proteins contained in the menstrual fluid were denatured in 4 M urea before sample processing and digestion. Because staphylococcal superantigenic toxins are resistant to proteolysis, several biochemical protocols were assessed for toxin digestion, including SDS-PAGE, followed by in-gel digestion [19], filter-aided sample preparation [20], and a rapid protocol combining protein reduction and alkylation in one step followed by a double digestion with LysC and trypsin (available as a commercial kit; see Section 4). From these preliminary tests, the rapid procedure was found to provide the most efficient digestion of the staphylococcal superantigenic toxins while also facilitating the detection of signature peptides, thus increasing the sensitivity of the toxin assays (Figure 1a, supplementary Figure S1).

Menstrual fluid is a very complex matrix that generates an overloaded peptide background (Supplementary Figure S2). To avoid interference during data acquisition, the LC gradient and SRM analysis were optimized to select the most responsive peptides producing the best transitions (see Section 4). In total, eight signature peptides (in their labeled and unlabeled versions) were included in the final LC-SRM method. For each of the peptides selected, three fragment ions were listed, thus resulting in a total of 48 SRM transitions (Figure 1b).

### 2.2. Investigation of Clinical Samples

Menstrual fluids were extracted from tampons collected either from healthy women or from patients with mTSS. The presence of *S. aureus* in the menstrual fluids and their ability to produce TSST-1, SEA, SEC, and SED toxins was determined using DNA microarrays, as described in Materials and Methods. Fluids were classified in three groups as follows: (1) *S. aureus tst+* with mTSS (n = 6 patients), (2) *S. aureus tst+* without mTSS (n = 16 healthy women), and (3) *S. aureus tst-* without mTSS (n = 6 healthy women). Each sample was spiked with defined amounts of our quantification standards—full-length isotope-labeled toxins—before biochemical processing and LC-SRM analysis. Toxins were quantified based on the unlabeled/labeled signal ratio determined for the most responsive signature peptides and the best transition (quantifier transition) (see Section 4 and Supplementary Table S1). The detection and quantification results are shown in Table 1.

**Figure 2:**
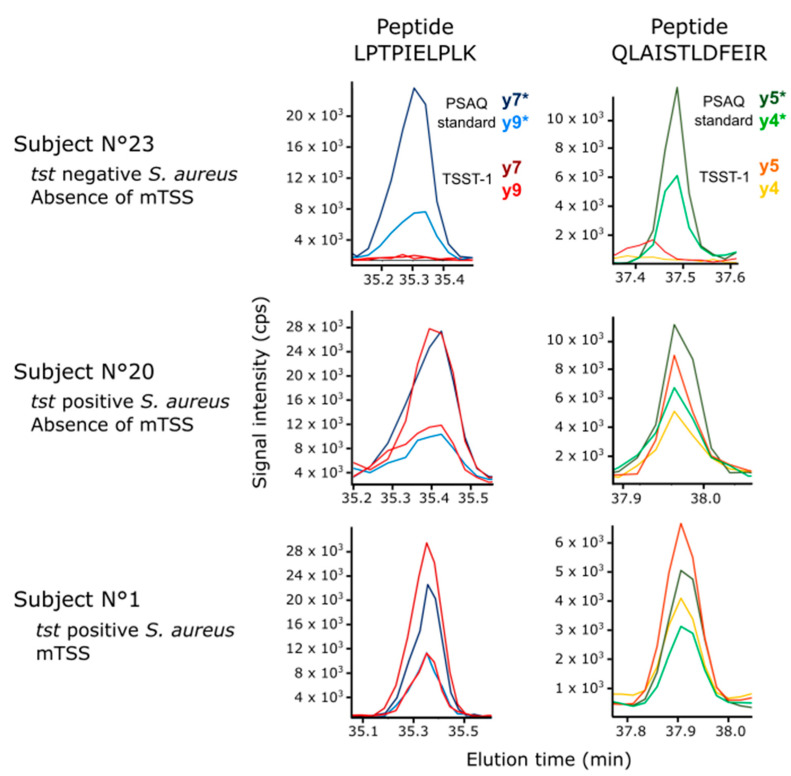
LC-SRM detection of TSST-1 in menstrual fluids. Extracted ion chromatograms obtained after protease-digestion of menstrual fluid, and analysis by scheduled LC-SRM. For greater clarity, the two most responsive signature peptides for TSST-1 and the two best transitions are shown. Red and orange traces correspond to SRM transitions monitored for endogenous TSST-1. Blue and green traces correspond to SRM transitions monitored for spike-in isotope-labeled TSST-1 (PSAQ standard). Y ions from isotope-labeled TSST-1 signature peptides are mentioned with an asterisk.

Using our method, TSST-1 and SEC were detectable in specimens of vaginal fluid containing *S. aureus* strains carrying the corresponding genes. Concentrations ranged from 0 to 0.46 µg/mL for TSST-1, and 0 to 1.07 µg/mL for SEC. TSST-1 was detected in 5 of the 6 samples from women with mTSS, and in 1 of 16 fluids collected from healthy donors with a *tst*+ vaginal isolate (Figure 2). The amount of toxin detected in the control sample was of the same order of magnitude as that observed in samples from patients with mTSS. SEC was detected in menstrual fluid from the patient with mTSS that had a *sec*+ isolate, but its concentration was too low to be quantifiable. It was also detected in one of the three menstrual fluid samples from heathy donors with *sec*-positive *S. aureus* (supplementary Figure S3). SEA and SED were never detected in clinical specimens, even though many *S. aureus* strains were positive for the corresponding genes. In our population, the detection of TSST-1 in the vaginal fluid was statistically well correlated with the occurrence of mTSS (Fisher test, *p* = 0.001; OR = 37.936, IC_95%_ = 2.735; 2419.686), not the SEA (*p* = 1) nor the SEC (*p* = 0.4) and SED (*p* = 1). No correlation was evidenced between the amount of TSST-1 detected in the vaginal fluid and the clinical or microbiological characteristics of the patients with mTSS.

## 3. Discussion

The goal of our work was to develop a mass-spectrometry-based proteomics workflow to quantify TSST-1, SEA, SEC, and SED in menstrual fluids to allow us to explore the pathophysiology of mTSS.

Recent interest in non-invasive diagnostic approaches to address women’s health issues, including infertility and uterine pathologies, has led to a search for methods to characterize menstrual fluid [22]. MS-based proteomics analysis can provide relevant information on the protein elements contained in menstrual fluids, which could be useful for histopathological research and for diagnostic purposes [10]. However, menstrual fluid is difficult to analyze due to its complexity and variations in its protein content. Consequently, very few articles have been published describing MS-based pathophysiology studies or assays to detect protein biomarkers [22]. Here, we harnessed the power of targeted MS-based proteomics to investigate the presence of staphylococcal superantigens produced in menstrual fluids from patients with mTSS, using menstrual fluid from healthy women as the control. Based on our previous experience in detecting SEs and TSST-1 in complex biological samples including serum [23], urine [19], and food [24], we developed a targeted proteomics workflow combining efficient biochemical preparation and quantitative LC-SRM to analyze menstrual fluids extracted from tampons used during menstruation. As expected, pre-analytical optimizations and analytical developments were challenging due to the variable composition of menstrual fluids, similar to food samples (Supplementary Figure S2) [24]. In addition, the presence of red blood cells, serum, uterine tissue, and vaginal secretions in the menstrual fluid extended the dynamic range of protein abundance beyond the 12 orders of magnitude described for serum or plasma matrices [11]. However, the combination of an efficient digestion protocol with an optimized LC-SRM analytical method ensured the sensitive detection of the toxins in clinical samples extracted from tampons. Our results confirmed the presence of TSST-1—at concentrations ranging between 10 ng/mL and 460 ng/mL—in menstrual fluid collected from 5 of the 6 women with mTSS. These results can be compared to two previous reports of vaginal detection of TSST-1 in mTSS. In 1987, Rosten and coworkers [16] developed an enzyme-linked immunosorbent assay (ELISA) to detect TSST-1 in vaginal washings. Their assay detected TSST-1 in clinical samples from 2 out of 9 patients with mTSS, only during the acute phase, with concentrations ranging from 2.2 ng/mL to 15.8 ng/mL. These results appear compatible with our results as vaginal washings are likely to be more diluted than menstrual fluids. In 2010, Schlievert and coworkers [25] used semi-quantitative western-blotting and compared signal intensities to TSST-1 standard curves to investigate the presence of TSST-1 in tampons collected from two women with mTSS. For these two patients, they reported TSST-1 levels of 69 and 80 µg per tampon (estimated tampon volume, 3 to 10 mL). These estimations are 20-fold higher than our results. It should be noted that these authors provided no information on the method used to quantify the purified TSST-1 used to create the standard curve. Our quantification results were obtained using AAA-calibrated isotopically-labeled standards, which have been demonstrated to be highly accurate. Consequently, differences in accuracy of the reference levels could explain the apparent discrepancies between the concentrations measured. In accordance with the results presented in these previous studies, we also detected TSST-1 in a sample from one healthy woman host to vaginal *tst*+ *S. aureus*. Thus, TSST-1 can be produced by *S. aureus* in the vagina during menstruation in the presence of a tampon without necessarily leading to mTSS. The pathophysiology of mTSS is complex [3]. The different steps for mTSS occurrence are the production of TSST-1 by *S. aureus* in the vaginal fluid, the passage of the toxin through the vaginal mucosa, and the activation of the immune system. In addition, natural antibodies neutralizing the toxin must be absent. MS-based detection of TSST-1 in menstrual fluids explores the first step of mTSS pathophysiology only. This explains why the detection of toxins in the vagina alone, as observed in one healthy woman, does not predict the occurrence of the disease. However, in our population, deliberately biased to include women with vaginal colonization by *S. aureus*, the detection of TSST-1 in the vaginal fluid was statistically well correlated with the occurrence of mTSS.

The level of TSST-1 production varied between women, underlining the importance of the appropriate use of intravaginal devices. No correlation was detected between the amount of TSST-1 detected in the vaginal fluid and the clinical or microbiological characteristics of the patients with mTSS. Interestingly, in one patient with mTSS, TSST-1 was undetectable by our method. There are numerous proteases within menstrual fluids [10] and protease inhibitors were not added to the menstrual fluids before storage. Possibly, toxins may have been degraded by proteases, especially TSST-1, which is less resistant to proteolysis than enterotoxins [10]. Based on this hypothesis, TSST-1 concentrations may be underestimated.

An issue that is not yet fully resolved is the almost exclusive association between TSST-1 secretion and the menstrual form of toxic shock [7]. Thanks to the multiplexing capabilities of targeted proteomics, we were able to simultaneously investigate the production of other staphylococcal superantigenic toxins in the vagina—including SEA, SEC, and SED—that have been associated with non-mTSS. In our cohort, SEA and SED were never detected in menstrual fluids, whereas SEC was detected in two clinical specimens, one collected from a patient with mTSS and one from a healthy volunteer whose *S. aureus* isolate was positive for *tst*, *sec*, and *sed*. Although co-secretion of TSST-1 could not be confirmed (due to signal contamination inducing changes in the relative intensity of SRM transitions), these results indicate that both TSST-1 and SEC can be produced by *S. aureus* in menstrual fluids, challenging the hypothesis that the epidemiological link between mTSS and the exclusive production of TSST-1 among staphylococcal superantigens is related to local conditions in the vagina, specifically inducing TSST-1 expression [1,26].

In the future, we plan to continue using the method presented here to explore toxin production in vivo in users of intravaginal devices to improve our understanding of the pathophysiology of mTSS and its prevention.

## 4. Materials and Methods

### 4.1. Ethical Review of the Study

This study was reviewed and approved by the local Ethics Committee (CPP Sud Est IV, Centre Léon Bérard, Lyon, France, N° L16-176). Written consent was obtained from all participants, or from the parents/guardians of participants under 18 years of age. 

### 4.2. Subjects

Healthy menstruating volunteers were recruited from March 2014 to June 2017 through the National Reference Center for Staphylococci (NRCS) though the gynecology departments of Hospices Civil de Lyon, and a national campaign including advertisement through social networks and other media. mTSS cases detected in France spontaneously referred to the NRCS were included in the study, as previous described [9,21]. mTSS diagnosis was based on the Centers for Disease Control and Prevention (CDC) diagnostic criteria. In this study, the selected patients correspond to the mTSS cases 2 to 7 described in the article by Jacquemond and coworkers [21].

### 4.3. Sample Collection and Microbiological Characterization

Tampons were collected as previously described [9,21]. Menstrual fluid was extracted from the tampon by soaking it in 15 mL of sterile distilled water and then pressing it. Fifty microliters of menstrual fluid were spread on a SAID chromogenic plate to selectively detect *S. aureus* (chromID™ *S. aureus*, Biomérieux, Marcy l’Étoile, France). Plates were incubated at 35 °C for 18–24 h under aerobic conditions. Suspicious colonies (pink to light pink) were identified by matrix-associated laser desorption ionization–time of flight (MALDI-TOF) mass spectrometry [21]. All *S. aureus* strains were genotyped using Identibac *S. aureus* Genotyping^®^ (Alere) DNA microarrays, as described previously [27]. Particular attention was paid to the presence of *tst*, *sea*, *sec*, and *sed* genes encoding TSST-1 and the enterotoxins SEA, SEC and SEC, respectively. A subset of 28 samples were selected from the collection of 737 samples previously described [9,21], 6 from patients with mTSS and 22 from healthy volunteers (Table 1). 

### 4.4. Production of Full-Length Stable Isotope-Labeled Toxins

Full-length stable isotope-labeled versions of TSST-1, SEA, SEC, and SED were synthesized as previously described [19,28]. Prior to their use as quantification standards (PSAQ standards), the stable isotope-labeled toxins were extensively purified and quantified by amino acid analysis (AAA) [29]. Isotope incorporation was verified by LC-MS and LC-SRM analysis, and was found to be greater than 99%.

### 4.5. Biochemical Preparation of Menstrual Fluids

The protein concentration of each menstrual fluid sample was determined using a bicinchoninic assay according to the manufacturer’s instructions (BCA Protein Assay kit, Sigma Aldrich, Saint-Quentin-Fallavier, France). A volume of menstrual fluid corresponding to 200 µg of proteins was spiked with defined and constant amounts of PSAQ standards for TSST-1, SEA, SEC, and SED. Then, the iST sample preparation kit (PreOmics) was used to prepare and digest menstrual fluid samples. Briefly, samples were alkylated and reduced for 10 min under agitation (1000 rpm) at 95 °C in a heating block. Then, samples were spun down in a centrifuge (300 rcf, 10 s) and digested with a trypsin/LysC mix (37 °C, 500 rpm, 4 h). The digested samples were transferred to a cartridge and washed to eliminate hydrophobic and hydrophilic contaminants. Purified peptides were eluted and dried in a vacuum evaporator.

### 4.6. LC-SRM Analysis

Dried peptide digests were solubilized in 25 μL of 2% acetonitrile, 0.1% formic acid. A 6-μL volume (equivalent to ≈7.5 µg protein) of this solution was analyzed by targeted proteomics. Targeted proteomics analyses were performed on a 6500 QTrap mass spectrometer (AB Sciex) operating in SRM mode. Liquid chromatography separation was performed on an Ultimate 3000 system (Dionex) equipped with a C18 Kinetex™ column (2.6 µm, 100 Å, 2.1 mm, 10 cm) using a two-solvent system with solvent A (2% acetonitrile, 0.1% formic acid) and solvent B (80% acetonitrile, 0.1% formic acid). Peptides were separated at a flow-rate of 60 µL/min over 40 min, applying a gradient from 4% to 30% solvent B in 28.5 min, and from 30% to 90% solvent B in 10 min. Mass spectrometry data were acquired in positive mode with an ion spray voltage of 4200 V; curtain gas was used at 30 p.s.i.; and the interface heater temperature was set to 250 °C. Collision exit, declustering, and entrance potentials were set to 20, 55, and 14 V, respectively. Scheduled SRM acquisitions (Table 2) were performed with Q1 and Q3 quadrupoles operating at unit resolution, and the acquisition time windows and target scan time were set to 420 s and 1.5 s, respectively.

### 4.7. LC-SRM Data Analysis

LC-SRM data was analyzed using Skyline software (version 21.1.0.278). All transitions were individually inspected, and were excluded if deemed unsuitable for quantification (low signal-to-noise ratio, obvious interference). Unlabeled/labeled peak area ratios were calculated for each SRM transition, and these ratios were used to determine the corresponding average peptide ratio. The TSST-1 concentration was calculated by averaging the ratio for each signature peptide.

## Figures and Tables

**Table 1:** Detection and concentration of superantigenic toxins in menstrual fluids collected from patients with mTSS and healthy women with *S. aureus* in their vaginal flora.

Subject Number	mTSS ^1^	Superantigenic Toxin Genes Present ^2^	LC-SRM Detection of TSST-1 ^3^	Detection of SEA, SEC, SED ^3^
1	Yes	*tst*, *sea*	460 ng/mL	Not detected
2	Yes	*tst*, *sea*	Not detected	Not detected
3	Yes	*tst*, *sea*	80 ng/mL	Not detected
4	Yes	*tst*, *sec*, *sed*	40 ng/mL	SEC detected but not quantifiable
5	Yes	*tst*, *sea*	70 ng/mL	Not detected
6	Yes	*tst*	10 ng/mL	Not detected
7	No	*tst*, *sed*	Not detected	Not detected
8	No	*tst*	Not detected	Not detected
9	No	*tst*, *sea*	Not detected	Not detected
10	No	*tst*	Not detected	Not detected
11	No	*tst*, *sea*	Not detected	Not detected
12	No	*tst*, *sea*	Not detected	Not detected
13	No	*tst*	Not detected	Not detected
14	No	*tst*	Not detected	Not detected
15	No	*tst*, *sea*	Not detected	Not detected
16	No	*tst*, *sea*	Not detected	Not detected
17	No	*tst*, *sea*	Not detected	Not detected
18	No	*tst*	Not detected	Not detected
19	No	*tst*, *sec*, *sed*	Not determined (signal contamination ^4^)	SEC 1.07 µg/mL
20	No	*tst*, *sea*	330 ng/mL	Not detected
21	No	*tst*	Not detected	Not detected
22	No	*tst*, *sea*	Not detected	Not detected
23	No	*sea*, *sed*	Not detected	Not detected
24	No	*sea*, *sed*	Not detected	Not detected
25	No	*sec*	Not detected	Not detected
26	No	*sec*	Not detected	Not detected
27	No	*sea*	Not detected	Not detected
28	No	*sea*	Not detected	Not detected

^1^ Clinical and biological characteristics of the cases of menstrual toxic shock syndrome included in the study are described in Jacquemond et al. [21]. Subject 1 corresponds to case 6, subject 2 to case 3, subject 3 to case 2, subject 4 to case 4, subject 5 to case 5 and subject 6 to case 7 of Table S1. ^2^ *tst*, gene coding for TSST-1; *sea*, gene coding for SEA; *sec* gene coding for SEC; *sed*, gene coding for SED. ^3^ When quantification was possible (i.e., signal/noise ratio >1/3 for the quantifier transition), TSST-1 and SEC concentrations in menstrual fluid are indicated. ^4^ Interferences and changes in the relative intensity and order of SRM transitions precluded the specific detection of TSST-1 (Supplementary Figure S3).

**Table 2:** Signature peptides and SRM transition parameters.

*S. aureus* Toxin	UniProt Reference	Signature Peptide ^1^	Fragment Ion	SRM Transitions ^2^		Collision Energy (eV)
				Q1 m/z	Q3 m/z	
TSST-1	P06886	LPTPIELPLK	+2y7	**560.9**	**809.5**	29.8
			+2y9 + 2	560.9	504.3	29.8
			+2y8	560.9	910.6	29.8
		LPTPIELPL [^13^C_6_,^15^N_2_] K	+2y7	**564.9**	**817.5**	29.8
			+2y9 + 2	564.9	508.3	29.8
			+2y8	564.9	918.6	29.8
		QLAISTLDFEIR	+3y5	**469.3**	**679.3**	25.7
			+3y4	469.3	564.3	25.7
			+3y3	469.3	417.2	25.7
		QLAISTLDFEI [^13^C_6_,^15^N_4_] R	+3y5	**472.6**	**689.3**	25.7
			+3y4	472.6	574.3	25.7
			+3y3	472.6	427.2	25.7
		HQLTQIHGLYR	+3y4	455.9	508.3	25.2
			+3y5	455.9	645.4	25.2
			+3y6	455.9	758.4	25.2
		HQLTQIHGLY [^13^C_6_,^15^N_4_] R	+3y4	459.3	518.3	25.2
			+3y5	459.3	655.4	25.2
			+3y6	459.3	768.4	25.2
SEA		NVTVQELDLQAR	+2y8	693.4	972.5	33.8
			+2y7	693.4	844.5	33.8
			+2y6	693.4	715.4	33.8
		NVTVQELDLQA [^13^C_6_,^15^N_4_] R	+2y8	698.4	982.5	33.8
			+2y7	698.4	854.5	33.8
			+2y6	698.4	725.4	33.8
		YNLYNSDVFDGK	+2y8	717.8	881.4	34.7
			+2y7	717.8	767.4	34.7
			+2y6	717.8	680.3	34.7
		YNLYNSDVFDG [^13^C_6_,^15^N_2_] K	+2y8	721.8	889.4	34.7
			+2y7	721.8	775.4	34.7
			+2y6	721.8	688.3	34.7
SEC		NLYEFNSSPYETGYIK	+2y8	**963.0**	**970.5**	43.5
			+2y5	963.0	581.3	43.5
			+2y8 + 2	963.0	485.7	43.5
		NLYEFNSSPYETGYI [^13^C_6_] K	+2y8	**966.0**	**976.5**	43.5
			+2y5	966.0	587.3	43.5
			+2y8 + 2	966.0	488.8	43.5
SED		STGDQFLENTLLYK	+2y7	814.9	880.5	38.2
			+2y6	814.9	751.4	38.2
			+2y5	814.9	637.4	38.2
		STGDQFLENTLLY [^13^C_6_] K	+2y7	817.9	886.5	38.2
			+2y6	817.9	757.4	38.2
			+2y5	817.9	643.4	38.2
		NVTVQELDAQAR	+2y8	672.4	930.5	33.0
			+2y7	672.4	802.4	33.0
			+2y5	672.4	560.3	33.0
		NVTVQELDAQA [^13^C_6_] R	+2y8	675.4	936.5	33.0
			+2y7	675.4	808.4	33.0
			+2y5	675.4	566.3	33.0

^1^ The unlabeled and isotopically-labeled versions are indicated for each signature peptide. ^2^ For each signature peptide, three transitions were selected and monitored. The transition generating the most intense signal was selected as the quantifier transition (in bold) and was used to determine each toxin’s concentration in menstrual fluid.

## Data Availability

The list of LC-SRM analyses, the corresponding raw data (.wiff files), and Skyline files (.sky files) have been deposited in the PeptideAtlas SRM Experiment Library (PASSEL) under dataset identifier PASS02786 (password EA7354n).

## Supplementary Materials

The following supporting information can be downloaded at: https://www.mdpi.com/article/10.3390/toxins14120886/s1, Figure S1: Comparison of the filter-aided sample preparation (FASP) and the PreOmics protocol for the preparation of menstrual fluid and LC-SRM detection of TSST-1, SEA, SEC and SED; Figure S2: SDS-PAGE analysis of menstrual fluids collected from healthy women or from patients with mTSS; Figure S3: Extracted ion chromatograms obtained for TSST-1 and SEC signature peptides after LC-SRM analysis of the menstrual fluid collected from subject 19 (*tst*, *sec* and *sed* positive *S. aureus*; absence of mTSS). Table S1: Estimation of Lower Limit Of Detection (LLOD) based on the analysis of zero samples.
