# Supplementary material for: Targeted Proteomics Analysis of Staphylococcal Superantigenic Toxins in Menstrual Fluid from Women with Menstrual Toxic Shock Syndrome (mTSS)
Source: Toxins (Basel). 2022 Dec 19;14(12):886. doi: 10.3390/toxins14120886 (PMC9788610; doi:10.3390/toxins14120886)
Supplement: Supplementary file 1 [file toxins-14-00886-s001.zip › toxins-2017348-supplementary.pdf]

Supplementary Materials: Targeted proteomics analysis of staphylococcal superantigenic toxins in menstrual fluid from women with menstrual Toxic Shock Syndrome (mTSS)

Marie Courçon, Cédric Badiou, Mathilde Louwagie, Sibyle Etievant, Michel Jaquinod, Gérard Linaand Virginie Brun

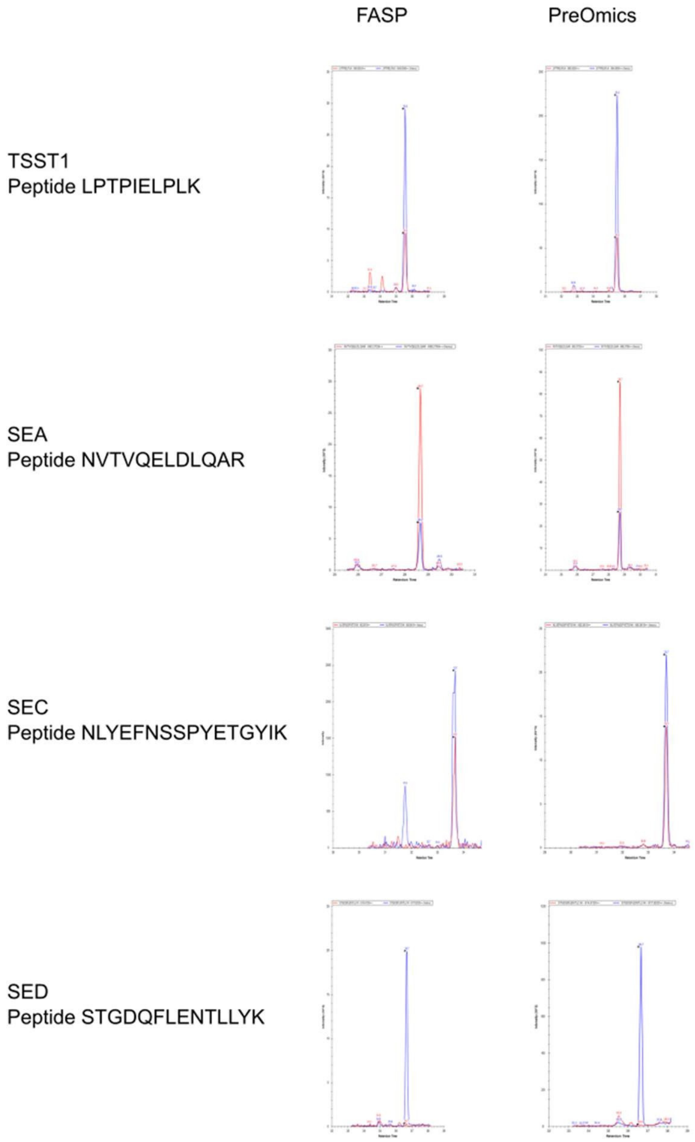

Figure S1. Comparison of the filter-aided sample preparation (FASP) and the PreOmics protocol forthe preparation of menstrual fluid and LC-SRM detection of TSST-1, SEA, SEC and SED. A menstrual fluid was spiked with unlabeled (red signal) and full-length isotope-labeled (blue signal) versions of the four toxins. The sample was processed with the two biochemical protocols and analyzedusing LC-SRM.

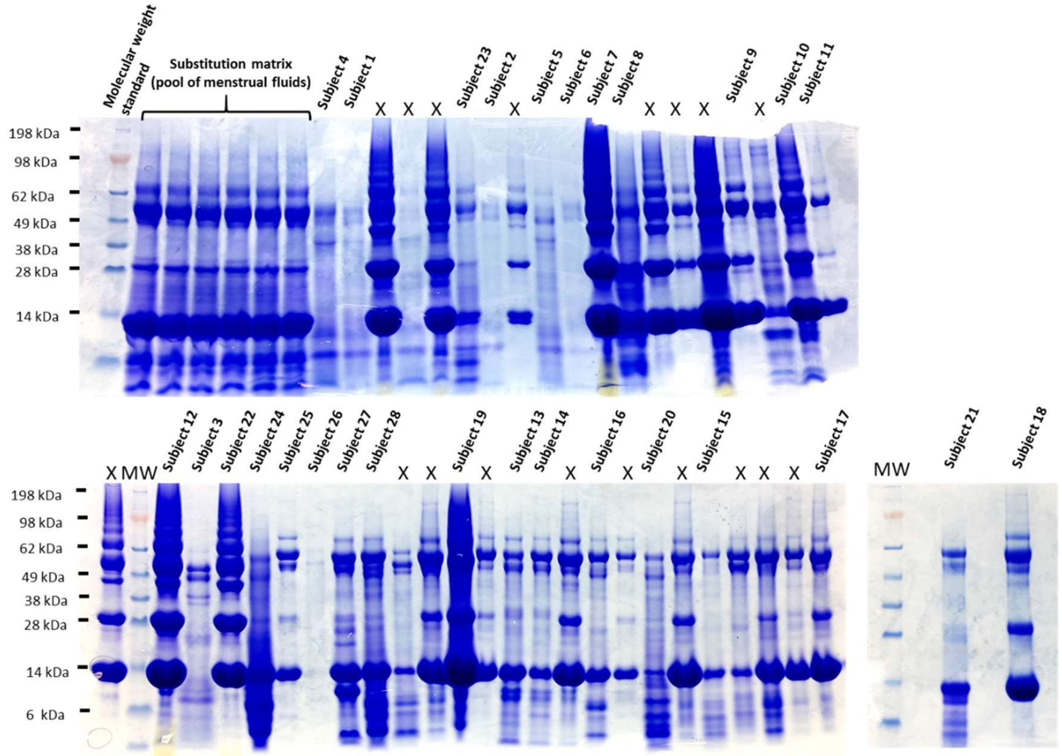

Figure S2. SDS-PAGE analysis of menstrual fluids (1 µl) collected from healthy women or from patients with mTSS.

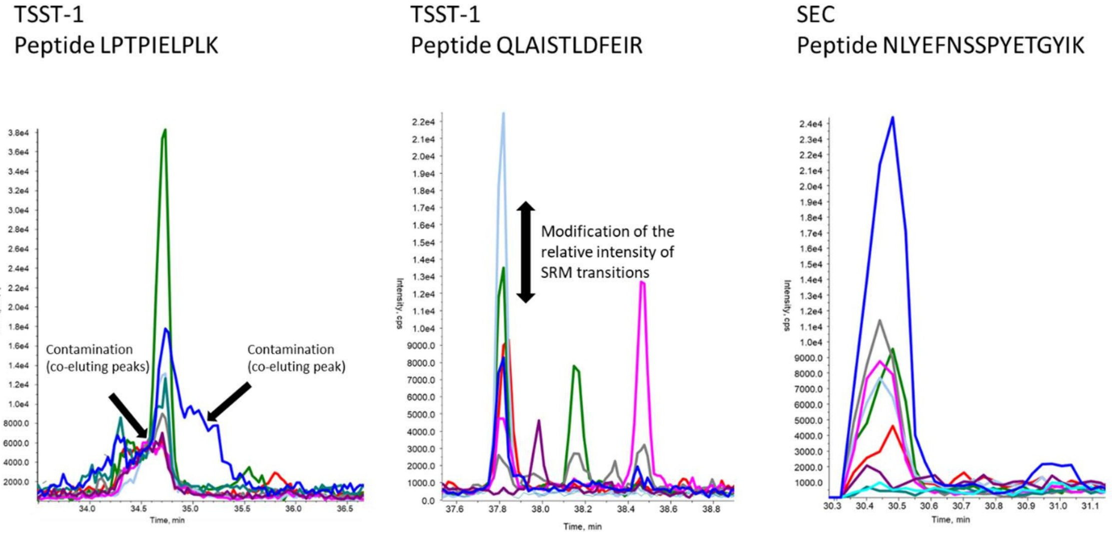

Figure S3. Extracted ion chromatograms obtained for TSST-1 and SEC signature peptides after LC- SRM analysis of the menstrual fluid collected from subject 19 (*tst, sec* and *sed* positive *S. aureus*; absence of mTSS). The SRM transition signals for two TSST-1 signature peptides could not be used forquantification due to multiple interferences. The SEC signature peptide was used to quantify the toxinin the menstrual fluid.

Table S1. Estimation of Lower Limit Of Detection (LLOD) based on the analysis of zero samples.

Three zero samples were created by adding fixed amounts of isotope-labeled TSST-1, SEA, SEC and SED (final concentration: 1 μg/mL) in a pool of menstrual fluids devoid of endogenous toxins. These samples were processed in parallel using the PreOmics pre-analytical protocol and were analyzed using the scheduled LC-SRM method applied to the clinical samples. LLOD was estimated for each signature peptide based on a signal-to-noise ratio > 3.

S. aureus **toxin**	**Signature peptide**	**Estimated LLOD**
TSST-1	LPTPIELPLK	30 ng/µl
	QLAISTLDFEIR	26 ng/µl
	HQLTQIHGLYR	27 ng/µl
SEA	NVTVQELDLQAR	19 ng/µl
	YNLYNSDVFDGK	21 ng/µl
SEC	NLYEFNSSPYETGYIK	59 ng/µl
SED	STGDQFLENTLLYK	25 ng/µl
	NVTVQELDAQAR	22 ng/µL
